# Neuroprotective Efficacy of Europinidin in Streptozotocin-Induced Memory Impairment by Modulation of Oxidative Stress, Inflammatory Mediators, and Cholinesterase Activity in Rats

**DOI:** 10.1155/2023/5248127

**Published:** 2023-01-31

**Authors:** Aftab Ahmad

**Affiliations:** ^1^Health Information Technology Department, Faculty of Applied Studies, King Abdulaziz University, Jeddah, Saudi Arabia; ^2^Pharmacovigilance and Medication Safety Unit, Center of Research Excellence for Drug Research and Pharmaceutical Industries, King Abdulaziz University, Jeddah, Saudi Arabia

## Abstract

**Materials and Methods:**

Oral acute toxicity studies were performed to evaluate the toxicological effects of europinidin in animals. In this study, four different animal groups (*n* = 6) were used. Group I was the normal control, group II was the STZ-induced diabetes control, group III was STZ + europinidin-treated (10 mg/kg), and group IV was STZ + europinidin-treated (10 mg/kg). The efficacy of europinidin at a dose of 10 mg/kg and 20 mg/kg was studied with single-dose administration of streptozotocin, which experimentally induced memory impairments in Wistar male rats for 38 days. The mean body weight and blood glucose levels were recorded at the initial and end of the study. The two behavioural paradigms (Y-maze and Morris water maze) were performed to evaluate spatial and working memory in rats. The biochemical parameters such as acetylcholinesterase, choline acetyltransferase, superoxide dismutase, glutathione transferase, malonaldehyde, catalase, and nitric oxide level as hallmarks of oxidative stress were measured. Additionally, the proinflammatory parameters were also determined to evaluate the neuroinflammatory responses associated with streptozotocin such as tumor necrosis factor-alpha (TNF-*α*) interleukin-1*β* (IL-1*β*), interleukin (IL-6), nuclear factor-kappa B (NF-*ƙ*B), interleukin (IL-10), and nuclear factor-erythroid factor 2-related factor 2 (Nrf2) in the perfused brain.

**Results:**

The rats in the europinidin-treated group exhibited a significant restoration of body weight and blood glucose level as compared with the streptozotocin control group. Furthermore, europinidin significantly modulated the spatial and working memory in rats, when assessed through behavioural paradigms. Streptozotocin caused a significant alteration in biochemical, neuronal enzymatic, and neuroinflammatory parameters, which were significantly restored to normal levels by europinidin.

**Conclusion:**

The present study attributed the neuroprotective efficacy of europinidin in experimental animal models by subsiding the several biomarkers of oxidative stress, neuroinflammation, and neuronal enzymatic activities.

## 1. Introduction

Alzheimer's disease (AD) is a most prevalent neurological disorder that is recognized to yield a chain reaction of changes in the brain and sociobehavioural interaction [1, 2]. Dementia relates to loss of memory and some other intellectual capacity that affects daily tasks. The frequency of dementia is growing globally, and it has become a serious societal concern as the older population has grown [3]. Dementia is recognized as both physiological aging and pathological disorders of the nervous system. It occurred due to a number of diseases and injuries that affect the brain, including Alzheimer's disease and/or strokes, which ultimately cause memory and learning impairment. Most people with dementia have Alzheimer's disease. Its most prominent clinical indications are gradual decreases in memory and cognition, which are accompanied by mental symptoms and aberrant behaviour. Previous studies revealed that Alzheimer's disease primarily affects those over the age of 65. As per 2017 data, approximately 46 million people are suffering from Alzheimer's disease globally [4, 5]. Acetylcholine (ACh), neuropeptides, serotonin, GABA (gamma-aminobutyric acid), estrogen, and nitric oxide are just a few of the neurotransmitters and neuromodulators that exhibit significant roles in cognitive functions [6]. The degree of cognitive function is influenced by brain cholinergic activity [7]. Cognitive deficits are resulted from decreased cholinergic functions. Several anticholinesterase medications have been claimed to ameliorate Alzheimer's disease by augmenting acetylcholine concentrations in the brain [8]. ACh governs several processes in the nervous system, including attention, arousal, and aggression [9]. ACh affects the striatum, hippocampus, and amygdala [10]. The basal forebrain and hippocampus both play important roles in memory and learning [11]. Studies based on oxidative hypotheses discovered a relationship between neurodegeneration and oxidative stress accompanying Alzheimer's disease [12]. Previous studies linked the relationship between the rate of ROS destruction and the ability of the body to remove ROS, which happened as a consequence of oxidative damage that induces tissue injury [13, 14]. Previously reported studies revealed the significance of several important brain areas known to exhibit cognitive effects and require antioxidants as essential nutritional supplements [15]. Previous data demonstrated that the neurodegeneration process might also include inflammation of neurons, an increase in ROS, which results in oxidative damage, and biochemical alterations in the brain that ultimately cause neuronal death [16]. According to research, free radical production generates oxidative stress, which can damage macromolecules and may be a key component in the onset of neurological problems [17]. Streptozotocin (STZ) is a naturally occurring antibiotic that has cytotoxic properties. Previous data postulated that STZ intracerebroventricular (ICV) administration causes a decline in cognitive functions [18, 19]. It triggers tau protein accumulation as well as altered cholinergic function, which is involved in memory impairment [20]. This cognitive loss might be related to a reduction in the expression of glycogen synthase kinase. There is a modest improvement in cognitive and global dementia measures provided by currently available medications [21]. As a result, there is a high demand for medications that combat the processes of aging, notably the deterioration in memory and cognitive functioning. Furthermore, the current treatment regimen, which comprises donepezil, rivastigmine, and galantamine, has some undesirable side effects such as nausea, vomiting, and loss of appetite. Hence, there is a need for alternative treatment options of natural origin with improved therapeutics. Flavonoids, a type of polyphenol of natural origin, are an important dietary constituent, which is very well known for their ability to modulate various health features [22]. They have become an integral part of herbal remedies, supplements, pharmaceuticals, and cosmetics due to their multiple physiological benefits against carcinogenicity, inflammation, redox imbalance, and neuronal damage [23, 24]. There is a wealth of research demonstrating the neuroprotective effects of flavonoids [24]. Their effectiveness in several neurological illnesses, such as Alzheimer's disease and Parkinson's disease (PD), has recently gained special attention. The neuroprotective properties of flavonoids offer various pharmacological properties, including anti-inflammatory, antiapoptotic, and antioxidant properties via several cellular pathways [23]. The molecules must cross obstacles such as numerous first-pass metabolic barriers, intestinal barriers, and eventually the blood-brain barrier as per the data suggested through several in vivo investigations. Data suggests that flavonoids have different pharmacokinetic profiles, which are linked to their pharmacodynamic characteristics. As a result, the brain permeability of flavonoids is currently being debated [25]. In plants, anthocyanidins are the sugar-free counterparts of anthocyanins. The flavylium ion, in which hydrogen atoms have been substituted with various groups, used to form these compounds. As a function of pH, they usually change color from red to purple, blue, and bluish-green [26]. A new anthocyanidin and pulchellidin found in the petals of *Plumbago pulchella*, while europinidin was found in the petals of *Plumbago europaea*. Both are monomethyl and 5,3'dimethyl-delphinidin [27]. Several flavonoids from medicinal plants have indeed been discovered as possible leads having the requisite pharmacological and molecular qualities to be prospective candidates for the prevention and treatment of Parkinson's disease. 8-Prenylnaringenin, europinidin, catechin gallate, homoeriodictyol, capensinidin, and rosinidin are among the flavonoids most commonly found in the plant [28].

**Figure 1:**
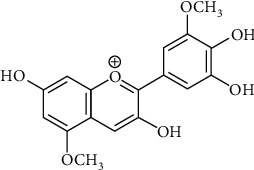
Chemical structure of europinidin.

Europinidin (Eu) is a water-soluble plant dye that is a derivative of delphinidin and blueish-red in color. It is considered to be an important anthocyanidin containing an O-methyl ring [29, 30], and the detailed chemical structure of europinidin is shown in Figure 1. The present study is aimed at evaluating the neuroprotective effect of europinidin in streptozotocin-induced memory impairment in experimental animal models.

## 2. Materials and Methods

### 2.1. Animals

In a standard laboratory setting, Wistar rats (adult male), weighing 160-200 g, were selected for the current study and kept at room temperature (24 ± 3°C) with a humidity control of 50-60%, following standard protocols. During the protocol, rodents were fed with pellet food and water ad libitum. The study protocol was carried out according to the institutional ethical guidelines on animal care (IAEC No-TRS/PT/021/009).

### 2.2. Drugs and Chemicals

The peptide antibiotic streptozotocin (STZ) (Sigma-Aldrich) was used in this study. Europinidin (an isolated compound from *Plumbaginaceae*) was obtained as gift sample from SRL, India. Commercially, available estimation kits (Modern Lab, M.S. India) were used to measure proinflammatory cytokines (IL-6, IL-1*β*, and TNF-*α*, Sigma-Aldrich). The analytical grade reagents and chemicals were utilized in the present study to carry out various evaluations.

### 2.3. Acute Toxicity Studies

Following OECD ANNEX-423 criteria, europinidin was tested for acute oral toxicity (LD50). Europinidin was dissolved in a sodium carboxyl methylcellulose (0.5 percent w/v) solution, which was given orally to rats (2000 mg/kg, body weight) as per the previously reported toxicity studies [31].

### 2.4. Experimental Design

The proposed study scheme of the experiment is based on previously reported methodologies with minimal modifications [32–34]. Postrandomization, rodents were segregated into four groups (*n* = 6) mainly normal control (group I), STZ control (group II), STZ + europinidin-treated 10 mg/kg (group-III), and STZ + europinidin-treated 20 mg/kg (group IV). All the animals were kept on 7-day acclimatization under conventional laboratory settings. A single dose of STZ (60 mg/kg, i.p.) was injected in 0.1 M cold citrate buffer (pH 4.5) into rats on the 8^th^ day of the protocol after fasting overnight for 12 hours beforehand [35]. On the 8^th^ day post-one-hour STZ injection, the treatment schedule was started, which was considered as day one of the protocol, and continued thereafter for the next 30 days with vehicle (2 mL/kg) for vehicle-treated and STZ control groups. Similarly, groups 3 and 4 received europinidin (10 mg/kg) and europinidin (20 mg/kg) daily single-dose administration for the next 30 days. To assess biochemical changes following dosing schedule completion, animals were subjected to behavioural testing before the brains of all animals were excised and preserved for further biochemical analysis. During the investigation, every attempt was taken to inflict as minimal suffering on the experimental animals as possible. Both the fasting blood glucose level (BGL) and body weight were monitored before and after the experiment.

### 2.5. Serum and Tissue Homogenate Preparation

The blood was collected from retro-orbital sinuses and transferred immediately into ice-cold 10 mL sterile test tubes. The serum was separated by carryout centrifugation of the obtained blood for five minutes at 1,200 × g and room temperature. The hippocampus region of the brain of the rats was removed and placed in liquid nitrogen, homogenized in 50 mM, pH 7.4, and ice-cold phosphate buffer solution, and centrifugation was performed for 20 minutes, at 7000 RPM at 4°C, and supernatant produced was used to determine tissue parameters.

### 2.6. Behavioural Tests

#### 2.6.1. Y-Maze Test

Y-maze consists of three arms (A, B, and C), which are adjusted at an angle of 120°, and made up of black acryl. In the procedure, animals were subjected to training by placing rats between intersecting three arms. The rat was allowed to explore the three arms for 3 minutes. In all the arms, the rats were allowed to roam around and were replaced. Following the training period, the animals were given the treatment drug, and the test was repeated. When all of the paws were on the floor of the arms, the total number of entries into the arms was recorded. When the rat enters the same arm in alternating order, the rat's successive entrances into each arm were measured. Total entries were also counted to assess the cognitive performance of disease control and treated rats [36, 37].

#### 2.6.2. Morris Water Maze Test (MWM)

Morris water maze (MWM) tests can be used to assess rodents' memory and learning capabilities. This apparatus comprises a circular pool surrounded by cues (120 × 60 cm diameter and deep) that are alienated into four equal quadrants in four directions. The water temperature of the pool was set all-time at (25 ± 1°C), and to make it opaque, white inert liquid was employed. A 10 cm circular platform was put 1 cm underneath the water's surface at the quadrant's central location. The MWM test comprises four days of training sessions comprising of time-bound 4 trials/day by 30 sec., the gap between two trials. We allowed rodents to swim freely in the water bath for 60 seconds (days 1 through 5) until they discovered the hidden platform in the water bath.

The participants were gently urged to find the platform and given 20 seconds to make sure they found it before they were removed. It remains in the same location during the course, but the starting positions are randomly selected. To determine how well the rats learned the platform, we measured their average escape latency times (ELTs).

Additionally, during the 60-second probe trial test, the water was withdrawn from the platform on the sixth day of the trial. A measure of spatial memory was determined by assessing the average time spent in the target quadrant, a measure of the number of times the target quadrant was entered, and an indicator of how late the target quadrant entered the target quadrant [38, 39].

### 2.7. Biochemical Estimation

#### 2.7.1. Acetylcholinesterase (AChE) Activity

The activity of acetylcholinesterase (AChE) in the hippocampus and serum was measured using the technique of [40] with acetylthiocholine (ATC) as the reagent. The testing mixture includes 0.4 mL (20%) of either plasma or brain homogenate, to which 100 *μ*L DTNB and 0.1 M and 2.6 mL (pH 8.0) phosphate buffer were added to the solution. The testing solution was mixed with bubbled air before being placed in the spectrophotometer. To establish a baseline value, the absorbance at 412 nm was measured after a reaction has reached a steady state. The absorbance of the ATC mixture that was measured at zero time and then after 10 minutes at 25°C after 5.2 *μ*L of ATC was introduced to this cuvette. AChE activity was measured in micromoles per minute per gram of brain or micromoles per minute per mL of plasma [40].

#### 2.7.2. Choline Acetyltransferase (ChAT) Activity

To test ChAT activity in the brain and plasma, rats were sacrificed; their heads were immediately submerged in liquid nitrogen for eight seconds. The skull was opened, the brain was removed, and the hippocampus was dissected at a temperature of 0-4°C. Tissue samples were preserved at -80°C until they have been used to evaluate ChAT activity. The assay of choline acetyltransferase activity was carried out by radiometric method. The incubation of homogenized brain samples in a solution of acetyl-CoA, acetyl-CoA along with SPA, MgCl_2_, NaCl, choline chloride, physostigmine salicylate, albumin, Triton X-100, EDTA, and sodium phosphate buffer (pH 7.4), was carried out for 40–60 minutes at 37°C, then cooled. ChAT activity was assayed as per Fonnum's technique [41].

#### 2.7.3. Biochemical Parameters

A biochemical immunoassay was carried out using a kit supplied by Transasia Biomedical Limited, Mumbai, India. An immunoassay kit was employed in rats to assess chemicals associated with inflammation. These included anti-inflammatory cytokines (IL-10), as well as lipid peroxidation and other biomarker activities (TNF-*α*, IL-1*β*, and IL-6). In addition, lipid peroxidation and several endogenous biomarker activities were also measured, including glutathione (GSH), superoxide dismutase (SOD), catalase activity (CAT), and nitric oxide (NO). Estimation of neuroinflammatory biomarkers (NF-*ƙ*B and Nrf2, Sigma-Aldrich) was performed by employing the standard immunoassay kits. Marker concentrations were determined using standard curves and expressed in pg/mL protein. The estimating technique is detailed in booklets supplied with commercially available kits.

### 2.8. Statistical Analysis

Prism 5 for Windows was used to evaluate the data (version 5.02). The findings were tabulated as mean ± SEM. For evaluating the significance and plotting the differences between the variables among the two groups, a one-way analysis of variance (ANOVA) with Tukey's multiple comparison test was used except the MWM test used two-way ANOVA followed by Bonferroni test post hoc. Statistically significant results were those with *p* values less than 0.05.

## 3. Results

### 3.1. Acute Toxicity Study

The acute toxicity of flavonoids in rats is safe even at dosages of up to 2000 mg/kg. During the 14-day acute toxicity period, there were no clinical symptoms or morbidity. According to the results of the acute oral toxicity trial, 10 mg/kg and 20 mg/kg of europinidin were determined to be safe for the main study.

### 3.2. Mean Body Weight Analysis

Effect of europinidin on body weight in streptozotocin-induced memory impairment in rats.

**Figure 2:**
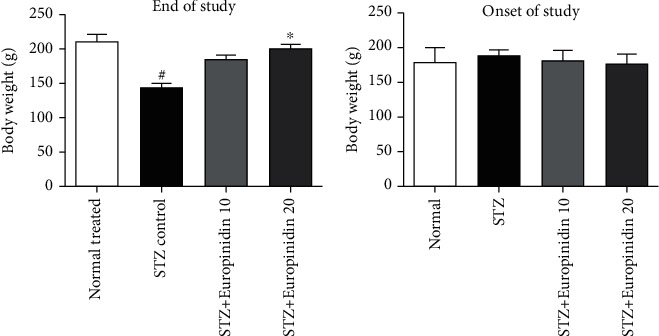
Mean body weight analysis. In the quantification of statistical data, the present study mean ± S.E.M (*n* = 6). A one-way analysis of variance, applied to Tukey's post hoc, shows statistically significant results in STZ-group vs. the control group at ^#^*p* < 0.01 and europinidin (20 mg/kg) vs. STZ-treated group (^∗^*p* < 0.05).

Figure 2 depicts the impact of europinidin on the bodyweight of rats with STZ-induced memory impairment, revealing that animals in the STZ control group had a subsequent drop in mean body weight (*p* < 0.01). The weight loss was significantly reversed in rats when a high dose of europinidin was administered (20 mg/kg) (*p* < 0.05). ANOVA and Tukey's post hoc analysis of europinidin (10 mg/kg) showed that the lower dose did not affect body weight parameters.

### 3.3. Blood Glucose

#### 3.3.1. Effects of Europinidin on Blood Glucose Levels

**Figure 3:**
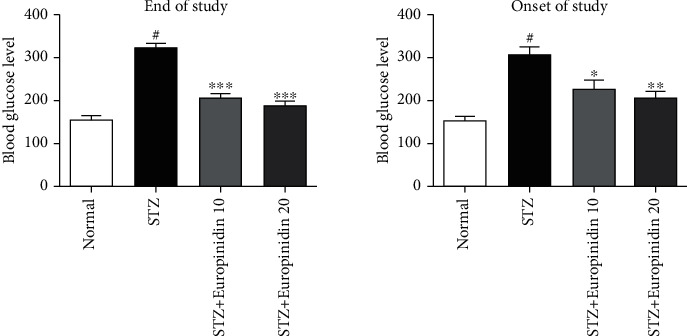
Streptozotocin-induced memory impairment in rats. Effects of europinidin on blood glucose levels. In the quantification of statistical data, the present study mean ± S.E.M (*n* = 6). A one-way analysis of variance, applied to Tukey's post hoc, shows statistically significant results in STZ group vs. the control group at ^#^*p* < 0.001 and europinidin (10 mg/kg) vs. STZ-treated group (^∗^*p* < 0.05; ^∗∗∗^*p* <0.001), also europinidin (20 mg/kg) vs. STZ-treated group (^∗∗^*p* < 0.01; ^∗∗∗^*p* <0.001).

The impact of europinidin on blood glucose levels in memory-impaired rats is visualized in Figure 3. When compared to control rats, STZ-induced animals had a substantial (*p* < 0.001) increase in BGL beginning on day 10. Furthermore, a one-way analysis of variance trailed by a post hoc test (Tukey's) resulted in remarkably restoring the blood glucose levels in the europinidin (10 and 20 mg/kg) group after 3 weeks of daily treatment compared to the STZ control group (*p* < 0.001).

### 3.4. Behavioural Paradigms

#### 3.4.1. Effects of Europinidin on the Y-Maze Test

**Figure 4:**
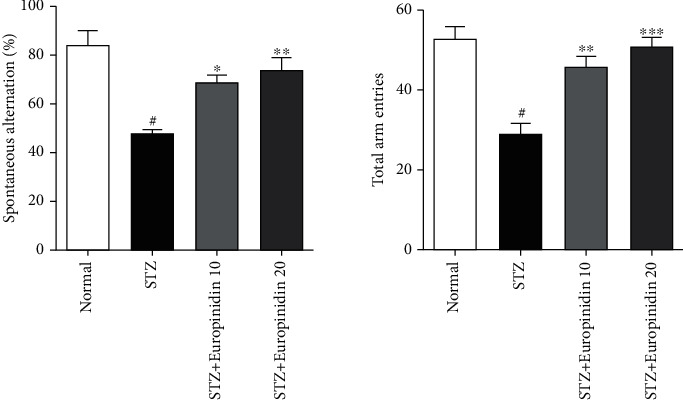
Streptozotocin-induced memory impairment in rats. Effects of europinidin on Y-maze test. Effects of europinidin on blood glucose levels. In the quantification of statistical data, the present study mean ± S.E.M (*n* = 6). A one-way analysis of variance, applied to the Tukey's post hoc, shows statistically significant results in STZ group vs. the control group at ^#^*p* < 0.001 and europinidin (10 mg/kg) vs. STZ-treated group (^∗^*p* < 0.05), also europinidin (20 mg/kg) vs. STZ-treated group (^∗∗^*p* < 0.001) during spontaneous alteration. Also, during total arm entries, statistically significant results were observed as the STZ group vs. the control group at (^#^*p* < 0.001), europinidin (10 mg/kg) vs. STZ-treated group (^∗^*P* < 0.05; ^∗∗^*P* <0.01), and europinidin (20 mg/kg) vs. STZ-treated group (^∗∗^*P* < 0.01; ^∗∗∗^*P* <0.001).

Figure 4 illustrated europinidin influence on the Y-maze test in streptozotocin-induced memory impairment. Rats were tested on their working memory through the Y-Maze test.

Our results showed that STZ-treated groups had a remarkably lower percentage of spontaneous alterations than control groups (*p* < 0.001). Furthermore, rats treated with an initial dosage of 10 mg/kg (*p* < 0.01) and a higher dose of 20 mg/kg (*p* < 0.001) substantially regulate the percent spontaneous change by restoring activity to the STZ-treated group. Comparing the STZ group with the control group, the STZ group had significantly fewer entries in each of the arms. By enhancing arm entries, the europinidin treatment group improved working memory significantly compared with the STZ group.

#### 3.4.2. Effects of Europinidin on Morris Water Maze Test

**Figure 5:**
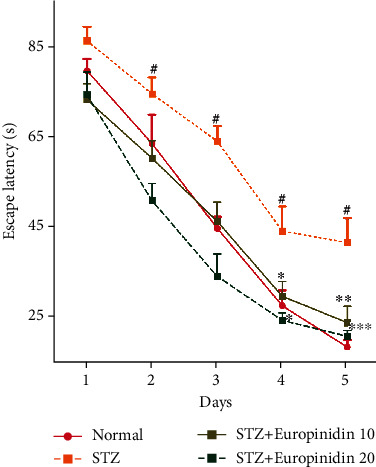
Streptozotocin-induced memory impairment in rats. Effects of europinidin on Morris water maze test. In the quantification of statistical data, the present study mean ± S.E.M (*n* = 6). A two-way analysis of variance, applied to the Bonferroni test post hoc, shows statistically significant results vs. the control group at #*p* < 0.05, ^∗^*p* < 0.05, ^∗ ∗^*p*<0.01 and ^∗ ∗ ∗^*p*<0.001.

Figure 5 shows the effect of europinidin on the MWM test in streptozotocin-induced memory impairment. Rats were tested on their spatial memory and learning skills using the MWM paradigm. In the experiment, all the animal groups were subjected to MWM test paradigms. The experiment exhibits two types of sessions: training sessions and test sessions. In the training session, all the animal groups showed decreased escape latency. Further, STZ induction significantly exhibits higher escape latency (*p* < 0.001) than normal treatment groups from day 2. Whereas, from day 2 onwards, europinidin-treated group dose-dependently reduced (*p* < 0.01 and *p* < 0.001) the latency time than that in the STZ control group.

### 3.5. Biochemical Analysis

#### 3.5.1. Effects of Europinidin on Acetylcholinesterase and Choline Acetyltransferase Levels

**Figure 6:**
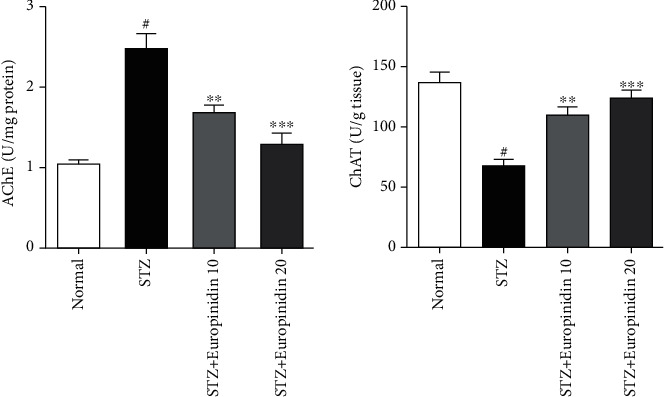
Streptozotocin-induced memory impairment in rats. Effects of europinidin on AChE and ChAT levels. In the quantification of statistical data, the present study mean ± S.E.M (*n* = 6). A one-way analysis of variance, applied to the Tukey's post hoc, shows statistically significant results in STZ group vs. the control group at ^#^*p* < 0.001 and europinidin (10, and 20 mg/kg) vs. STZ-treated group , ^∗∗^*p* < 0.01, and ^∗∗∗^*p* < 0.001) during different neuroenzyme estimations.

The effects of europinidin on acetylcholinesterase and choline acetyltransferase levels have been illustrated in Figure 6. The normal control group displayed significantly higher activity levels of AChE (*p* < 0.001) as compared to the STZ-intoxicated group. The AChE level in the treatment group dropped significantly (*p* < 0.001) relative to the normal control group after the europinidin treatment (10 mg/kg and 20 mg/kg). Thus, acetylcholine was broken down by europinidin treatment. Another study comparing STZ with the control group showed a significant decline in ChAT (*p* < 0.001). Europinidin significantly increased ChAT levels at both doses (*p* < 0.001) when compared to an STZ-intoxicated group.

#### 3.5.2. Oxidative Parameters

**Figure 7:**
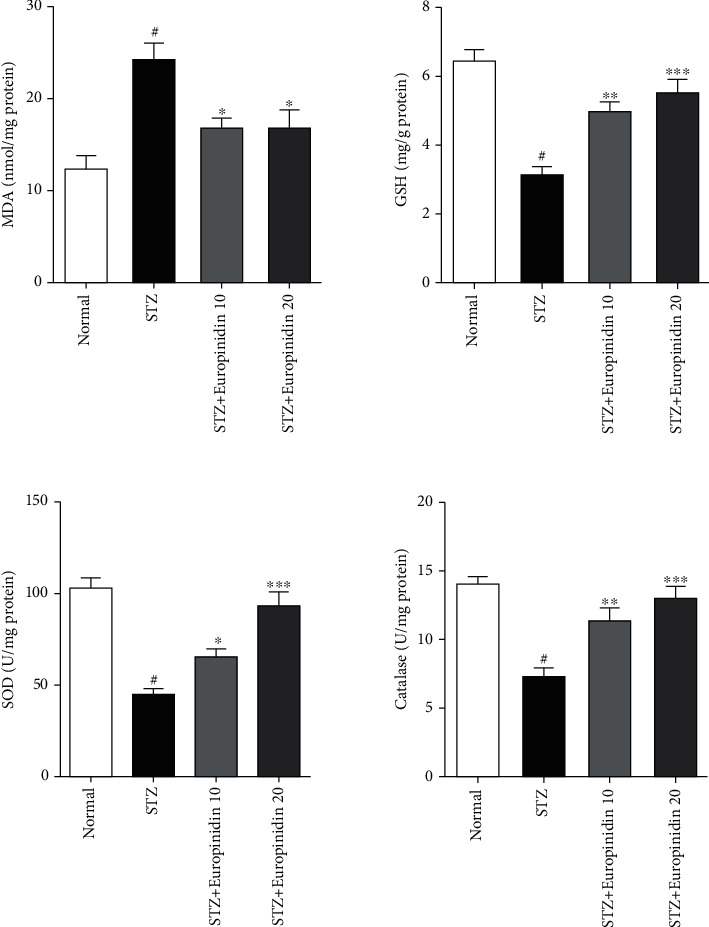
Streptozotocin-induced memory impairment in rats. Effects of europinidin on oxidative parameters. In the quantification of statistical data, the present study mean ± S.E.M (*n* = 6). A one-way analysis of variance, applied to Tukey's post hoc, shows statistically significant results in STZ group vs. the control group at ^#^*p* < 0.001 and europinidin (10 and 20 mg/kg) vs. STZ-treated group (^∗^*p* < 0.05, ^∗∗^*p* < 0.01, and ^∗∗∗^*p* < 0.001) during different biochemical estimations.

*(1) Effects of Europinidin on Oxidative Parameters*.  Figure 7 displays the influence of europinidin on the regulation of several endogenous oxidative parameters as a neuropathology marker. A more in-depth analysis revealed that STZ treatment group MDA levels increased significantly (*p* < 0.001) compared to control group levels. A STZ control group did not show any alterations in MDA levels at either 10 mg/kg or 20 mg/kg ((*p* < 0.05); (*p* < 0.001)) europinidin treatment. Another set of experiments evaluated the levels of GSH, SOD, and CAT and found substantial drops in all three parameters (*p* < 0.001) in the STZ control group than in the standard treatment group.

#### 3.5.3. Nitrite Estimation

**Figure 8:**
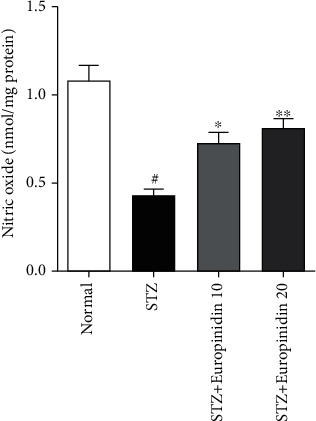
Streptozotocin-induced memory impairment in rats. Effects of europinidin on nitrite levels in the quantification of statistical data; the present study mean ± S.E.M (*n* = 6). A one-way analysis of variance, applied to Tukey's post hoc, shows statistically significant results in STZ group vs. the control group at ^#^*p* < 0.001 and europinidin (10 and 20 mg/kg) vs. STZ-treated group (^∗^*p* < 0.05 and ^∗∗^*p* < 0.01.

*(1) Effects of Europinidin on Nitrite Levels*.  Figure 8 highlights europinidin potential modification of endogenous nitric oxide levels. A comparison of STZ-treated and control groups showed that nitric oxide levels were remarkably abridged in STZ-treated groups. The treatment with europinidin at doses of 10 mg/kg (*p* < 0.05) and 20 mg/kg (*p* < 0.01) controlled nitric oxide levels more significantly than in the STZ control group.

#### 3.5.4. Estimation of Proinflammatory and Anti-Inflammatory Markers

**Figure 9:**
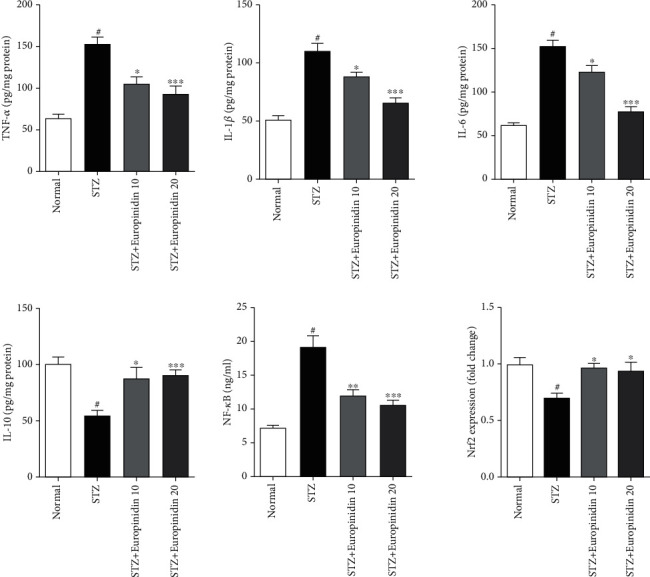
Streptozotocin-induced memory impairment in rats. Effects of europinidin on antiproinflammatory cytokines. In the quantification of statistical data, the present study mean ± S.E.M (*n* = 6). A one-way analysis of variance, applied to Tukey's post hoc, shows statistically significant results in STZ group vs. the control group at ^#^*p* < 0.001 and europinidin (10, and 20 mg/kg) vs. STZ-treated group (^∗^*p* < 0.05, ^∗∗^*p* < 0.01, and ^∗∗∗^*p* < 0.001) during different proinflammatory marker estimations.

*(1) Effects of Europinidin on Proinflammatory and Anti-Inflammatory Markers*.  Figure 9 displays europinidin potential roles in the regulation of endogenous proinflammatory indicators. A significant difference (*p* < 0.001) was noted between the STZ-treated and control groups in terms of various proinflammatory markers including TNF-*α*, IL-1*β*, IL-6, and NF-ƙB. A 10 mg/kg dose of europinidin resulted in a less significant drop (*p* < 0.05) in contrast to a 20 mg/kg dose of europinidin (*p* < 0.001) in proinflammatory biomarker levels. A series of experiments also found lower levels of anti-inflammatory markers such as IL-10 and Nrf2 in the STZ control group compared with the standard group (*p* < 0.001). A modest dosage of europinidin (10 mg/kg) resulted in a less significant modification of IL-10 (*p* < 0.05). An increased dose of europinidin (20 mg/kg) produces a significantly altered expression of IL-10 and Nrf2 as compared with the STZ control group (*p* < 0.001).

## 4. Discussion

The purpose of this study was to look into the possible neuroprotective efficacies of europinidin in rats with streptozotocin-induced memory impairment. Acute toxicity tests for europinidin were done in the assessment to determine any toxicities associated with europinidin. Europinidin at a dose of 10 and 20 mg/kg was determined to be safe in rats, with no adverse effects. In the current investigation, we found that the impairment in memory is remarkably caused by STZ [16]. Streptozotocin injections were shown to diminish brain energy consumption and disrupt cholinergic transmission by blocking insulin receptors in neurons from becoming activated [42]. Furthermore, we made a comparison between normal treated rats with STZ control rats; in that, STZ-treated group exhibits significant alterations, i.e., increase in blood glucose level and a significant reduction in body weight. Previously, reported studies established that alteration, i.e., increase in blood glucose level and reduction in body weight, is an important hallmark for the pathogenesis of Alzheimer's disease [43]. We include behavioural paradigms in the present study to elucidate spontaneous working memory and spatial memory in rats. The working memory of rodents has been evaluated through the use of a Y-maze behaviour test previously reported by the animal research community [44]. The percentage of rats returning to previously visited arms during a specific period was found to be a spontaneous activity. There is a significant reduction in spontaneous alteration, indicating behavioural toxicity linked with the STZ-treated group as opposed to the normal control group [45]. Furthermore, animals treated with europinidin significantly reinstate the memory function in the maze paradigm by refining the percentage of spontaneous alteration that enhances the cognitive function in rats. Similarly, we also employed another behavioural paradigm (MWM test) for memory alteration in animal models. Similar to previous observations, in the MWM test, STZ-treated group exhibits a notable falloff in the escape latency as a hallmark of spatial memory in rats [38]. Similarly, europinidin dose-dependently modulates the spatial performance via escape latency, which revealed its ability to subside the STZ-induced memory impairment in rodents.

Previous data suggested the critical role of the cholinergic system in the directive of several processes based on sensory functions including cognition, sleep, and arousal [46]. Furthermore, changes in the cholinergic system can result in memory loss and behavioural disorders. According to the literature, STZ treatment promotes cholinergic neuronal depletion in rats, which is associated with changes in cholinergic enzymatic activity such as higher levels of acetylcholinesterase (AChE) and a consequent reduction in choline acetyltransferase (ChAT) [42, 47, 48]. In the existing study, we detected that europinidin dose-dependently modulates cholinergic activities through a remarkable decrease in the AChE activity and subsequently improves the ChAT activity. Flavonoids exhibit AChE inhibitory effects due to their antioxidant potential, which is linked to enhancing the antioxidant defence mechanism of cholinergic neurons and thus alleviating behavioural and cognitive deficits.

Forgoing research postulated that free radical generation via oxidative stress leads to hindering normal brain antioxidant activities [49, 50]. In the present investigation, we observed that STZ administration causes remarkable alterations in several oxidative stress factors such as superoxide dismutase (SOD), glutathione transferase (GSH), malonaldehyde (MDA), and catalase whereas a significant reduction in nitric oxide levels. Similar to previously reported data, STZ administration causes decreasing in GSH, SOD, and catalase levels in the samples obtained from perfused rat brains. Whereas, it is also remarkably elevated in several oxidative biomarkers such as nitric oxide and MDA [51–54]. The antioxidant consequence of europinidin on STZ-activated oxidative damage has been demonstrated, with a dose-dependent reduction and restoration of antioxidant levels at normal levels. Moreover, europinidin treatment showed a significant decrease in the elevated levels of nitric oxide by subsiding NOS activity in the brain.

The reactive oxygen species greatly altered several endogenous molecular pathways including NF-*ƙ*B, which link to elevated levels of proinflammatory cytokines. According to earlier studies, STZ increases levels of cytokines associated with inflammation, including TNF-*α*, IL-1*β*, IL-6, and NF-*ƙ*B, and decreases levels of IL-10 and Nrf2 in rodents [51–54].

The limitations of the study are short duration with the use of minimum animals. We will plan for further studies to perform immunohistochemistry to confirm the mechanism of europinidin. However, europinidin could be considered an option in preclinical and clinical research for Alzheimer's disease.

## 5. Conclusion

For the first time, a study verified the ability of europinidin to treat oxidative stress, inflammation, and neurotoxicity and to improve biochemical markers of these disorders. In STZ-induced rats, europinidin enhanced spatial and cognitive learning behaviour. In STZ-induced neurotoxicity, europinidin exhibits strong antioxidant properties by subsiding the elevated levels of AChE enzymatic activity. Our outcomes publicized that dose-dependent administration of europinidin significantly modulates the above-mentioned parameters, which postulated the possible neuroprotective, anti-inflammatory, and antioxidant potentials of europinidin. This may prove to develop economical phytochemical alternatives for the prognosis of diabetes. The anti-inflammatory effect of europinidin in STZ-induced memory impairment demonstrated its ability to modulate several neuroinflammatory cytokines. The present study gives insights into the significance of europinidin in memory impairment that can possibly address its efficacy to penetrate BBB as a future direction for more budding researchers. Considering the above information, europinidin has the potential to be an effective natural component for treating neurodegenerative disorders.

## Data Availability

All data have been incorporated in the manuscript.
